# Review of Public Transport Needs of Older People in European Context

**DOI:** 10.1007/s12062-016-9168-9

**Published:** 2016-11-12

**Authors:** B. P. Shrestha, A. Millonig, N. B. Hounsell, M. McDonald

**Affiliations:** 1 Transport for London, Palestra House, 197 Blackfriars Road, London, SE1 8NJ UK; 2 Austrian Institute of Technology, Giefinggasse 2, 1210 Vienna, Austria; 3 Transportation Research Group, University of Southampton, Southampton, SO17 1BJ UK

**Keywords:** Older people, Public transport, Older people profile, Transport accessibility

## Abstract

People’s life expectancy is increasing throughout the world as a result of improved living standards and medical advances. The natural ageing process is accompanied by physiological changes which can have significant consequences for mobility. As a consequence, older people tend to make fewer journeys than other adults and may change their transport mode. Access to public transport can help older people to avail themselves of goods, services, employment and other activities. With the current generation of older people being more active than previous generations of equivalent age, public transport will play a crucial role in maintaining their active life style even when they are unable to drive. Hence, public transport is important to older people’s quality of life, their sense of freedom and independence. Within the European Commission funded GOAL (Growing Older and staying mobile) project, the requirements of older people using public transport were studied in terms of four main issues: Affordability, availability, accessibility and acceptability. These requirements were then analysed in terms of five different profiles of older people defined within the GOAL project – ‘Fit as a Fiddle’, ‘Hole in the Heart’, ‘Happily Connected’, An ‘Oldie but a Goodie’ and ‘Care-Full’. On the basis of the analysis the paper brings out some areas of knowledge gaps and research needed to make public transport much more attractive and used by older people in the 21st century.

## Introduction

People’s life expectancy is increasing throughout the world as a result of improved living standards and medical advances. In the EU, life expectancy at birth is projected to increase from 76.7 years in 2010 to 84.6 years in 2060 for males and from 82.5 years in 2010 to 89.1 years in 2060 for females (EC 2012). It is predicted that the EU population aged 65 and above will almost double by 2060, rising from 87.5 million in 2010 to 152.6 million (EC 2011).

The natural ageing process is accompanied by physiological changes which can have significant consequences for mobility. A list of age-related changes and their consequences for mobility have been identified by various researchers including Gewalt (Gewalt 2011). These changes include: Reduced flexibility and strength; impairment of visual perception; increased vulnerability to bone fracture; etc. In addition, Mollenkopf and Flaschenträger (2001) found that “almost all older persons, regardless whether they participate in walking, cycling, driving or using public transport, suffer from the tighter and more aggressive traffic”. By this, the authors’ are taken to mean increased traffic density and flow, as well as some drivers lack of consideration for other road users. These characteristics tend to affect older people more than other age groups. As a consequence, older people tend to make fewer journeys than other adults and may change their transport mode. Of course, a reduction in travel in older age groups is also simply because of a reduction for that age group of full time work. Mobilität in Deutschland survey (MiD 2008) found that with age, older people start to walk more, drive less and use more public transport. After 55, car usage was found to decrease constantly, whilst walking increased and public transport became a more used alternative for those aged 75 or older. In the course of the MOBILATE project it was also shown that there are different sub-groups of older people with different behaviour patterns and corresponding needs (Mollenkopf et al. 2005). The mobility of older people can therefore be strongly dependent on the provision and quality of public transport services, taking into account the heterogeneity of the target group.

Available, effective and affordable transport facilities provide access to people and places necessary to maintain a good quality of life (Metz 2003). Access to public transport can help older people to avail themselves of goods, services, employment and other activities. With the current generation of older people being more active than previous generations of equivalent age, public transport will play a crucial role in maintaining their active life style even when they are unable to drive. We should note here that whilst car ownership rates for older people are increasing (Follmer et al. 2009), car use decreases with age for older people, partly because of the increasingly challenging driving environment for them. Hence, public transport is important to older people’s quality of life, their sense of freedom and independence. This has been confirmed in an extensive survey in the UK (Gabriel and Bowling 2004) and features in the World Health Organisation’s checklist of essential features of age-friendly cities (WHO 2007).

In this context, the European Commission funded the GOAL (Growing Older and staying mobile) project, which included a review of the public transport needs of older people, and developed an action plan for addressing those needs. The process involved various surveys and the profiling of older people into one of five categories according to health and mobility characteristics. This paper describes the requirements of older people using public transport in terms of four main issues: Affordability, availability, accessibility and acceptability. The paper then presents the analysis in terms of five different profiles of older people to bring out areas of knowledge gaps that need to be understood and tackled to make public transport significantly better for older people.

This paper is based entirely on the results of the GOAL project, covering in particular the needs of specific profiles of older people and the related implications for public transport services. The results are based on utilisable information from projects and publications (in terms of accessibility, language and quality)^1^. The paper is structured in six sections, including the ‘Introduction’. Section 2 describes the changes in mobility patterns with age followed by the requirements of public transport for older people in Section 3. Section 4 analyses these requirements, with a fuller discussion of issues in Section 5 to bring out conclusions in Section 6.

## Mobility Patterns and Barriers

Changes in mobility patterns of older people are caused by different factors, depending on individual circumstances. Besides increasing physical or mental limitations, life-changing events play a major role in altering mobility behaviour and needs (GOAL D2.1, 2012). Retirement, illness and death of a partner / spouse / close relative are typical examples of such events which affect mobility needs and options. Other important events include divorce, marriage, children moving out and the birth of grandchildren. Such life-changing events and the related consequences for older people often result in changes in older people’s mobility patterns and needs, particularly regarding trip purpose (e.g. more health-related trips) as well as the spatial activity range and reachability of desired destinations (e.g. due to relocations of family, friends or older people themselves). With regards to the factors influencing older peoples’ mobility, Webber et al. (2010) conceptualized a framework for mobility that is based on seven life-space locations (based on distances), each of which is composed of mobility determinants related to cognitive, psychosocial, physical, environmental, and financial factors. Among these seven life-spaces, service community and surrounding area are the locations where public transport plays a role.

Travel purposes of older people revealed from a survey carried out in Germany (MiD 2008) showed that shopping and leisure are the main motives for travel of those over 60. Access to healthcare, food shops, post offices and other cultural, social and leisure facilities (including libraries, leisure centres, non-food shops, town centres and places of worship) were considered important in a review of local transport accessibility planning (Help the Aged 2006a, b). Of these, healthcare was overwhelmingly recognised as a key service to which access was important. Apart from trip purposes, other typical indicators such as frequency, distances and duration of trips are affected. Compared to younger groups, average trip lengths per day decrease constantly from the age of about 50 onwards. This can be linked to a reduction/removal of journey to work trips starting with retirement (Bakaba and Ortlepp 2010). A Belgian study also presented evidence that, on average, male senior citizens travel a longer distance daily than females.

The general frequency of trips (i.e. traveling directly between two destinations for a specific trip purpose, e.g. work, shopping) depends both on owning a driving license and the monthly income, amongst other factors. On average, daily travel time is 60–70 min with no conclusive findings concerning changes in average daily travel time with rising age. The average daily distance is about 29 km, with distances decreasing with rising age (60–69 years: 31.2 km, 70–79 years: 25.2 km, 80 and over: 25.2 km). Older people with a low income and/or without a driving license are more likely to have shorter travel distances (Christaens et al. 2009). Overall, travel costs become more important than travel time with growing age; since older people have more time and often less money they tend to choose alternatives that are cheaper but with longer travel times (Su 2007).

In many cases changes in mobility patterns can be linked to the decreasing ability of older people to overcome different barriers. These include physical, psychological and economic barriers to travel, including diminished motor, sensory and cognitive abilities for some (ECMT 2002). An Austrian study investigating the mobility patterns and barriers of 15 different groups of transportation disadvantaged people (e.g. people with physical or sensory disabilities, people at risk of poverty, single parents) showed that older people in particular experience barriers related to lack of accessibility to the physical environment, insufficient provision of information and inadequate public transport services (Sammer et al. 2013). All of these aspects specifically affect people who are dependent on the use of public transport services. For many of those no longer able to drive, public transport is an important alternative means of transport, although others can include Community Transport, Special Transport services, Para-transit-type services (i.e. special transportation services for people with disabilities, often provided as a supplement to fixed-route bus and rail systems by public transit agencies), taxis and lifts from family and friends. For further information on Demand Responsive Transport Systems worldwide readers are referred to Ambrosino et al. (2004).

Ceasing to drive provides a major challenge for many older people as it is often linked to emotional factors like fear of loss of personal freedom (Berg et al. 2015; Musselwhite and Shergold 2013; Musselwhite 2010). In order to keep the rapidly increasing population of older people actively involved in their daily activities, it is therefore vital that public transport facilities available are adequate to provide acceptable levels of mobility for the specific needs of older people (Gilhooly et al. 2002). This is not only important for the individual well-being of older people; it also provides significant economic return due to improved quality of life especially by supporting social engagement and physical activity, hence improving mental and physical health (Browning and Thomas 2013). To address this issue, it is essential to identify the requirements of public transport for older people depending on their circumstances.

## Requirements of Public Transport for Older People

The requirements of older people using public transport have previously been studied by considering them in groups with particular characteristics. Neugarten (1974) and Baltes (1997) distinguished older people in different age intervals and explored the needs for each of those intervals. In the Euro Access project, a differentiation was made based on the impairments of older people: Mobility impairments, visual impairments, hearing impairments and cognitive impairments. Apart from the user perspective, the needs of older people could be explored from a system perspective (i.e. what are the characteristics of a system suitable for older people).

To make public transport an attractive alternative for older people, all the elements of the public transport chain need to be considered. If any of these elements is unsuitable for them, they may not be able to use public transport at all, however excellent the remaining elements. These issues include: accessibility of bus, bus stop facilities, availability of information, ease of wayfinding, availability of toilets, etc. A recent European survey showed that regular users are concerned not only with the conditions on board the bus, but also with the ease of the entire trip (from its planning to its conclusion in a door-to-door logic). In particular they are sensitive to service quality, including personal security, reliability, service frequency, continuity of the service (temporal and physical), comfort, cleanliness, customer care, real time information and affordability of the fare.

There are various ways of looking at an ideal public transport system for older people. Earlier studies promoted an idea of 5 A’s of public transport need for older people: Availability, acceptability, accessibility, adaptability and affordability. Whereas other studies such as that by Borges (2012) listed the issues as: Accessibility, safety, affordability, availability and acceptability. A resource guide recently developed by the UK Department for Transport and the UK’s Passenger Transport Executive Group report (pteg 2010) gave the four main issues as: affordability, availability, accessibility and acceptability. This categorisation builds on the previous approaches and combines all originally identified aspects in these four categories. On this basis, the following subsections give a full description of the aspects covered in the categories, which will subsequently be used for discussing the needs of different profiles of older people regarding public transport.

### Accessibility

Accessibility is a key issue for older people using public transport; key issues are the location of relevant bus stops with respect to the trip origin and desired destination(s); the quality of the infrastructure supporting the walking segments of the trip; and the accessibility of the buses themselves (e.g. whether low-floor or not). Various studies have been carried out to examine the issues of older people and people with disabilities (e.g. UNDP (2010), MEDIATE (2008)). Despite the progress made in recent years in improving accessibility for all, it is estimated that 10–20 % of European citizens, including people with disabilities and older people, still experience barriers and reduced accessibility to transportation (Borges 2012).

The design of the bus itself can also be an important factor in its accessibility to older people who ideally need have a seat available and to be able to access it. Attributes of an ideal bus for older people include: stepless entrances (low floor, kneeling facility), handrails, priority seating facilities (in the front part), real-time audible information, wheelchair space, etc. Design should also accommodate people with difficulties other than being less mobile. For example, visually impaired people will appreciate colour contrasts within the bus (e.g. so that handrails are clearly visible)

Similarly, from an older person’s perspective, the attributes of an ideal bus stop include being close to their residence, located at a visible and well-lit place, information provision (preferably real time), clean and with weather protection, provision of seating facilities and help point provision. Poor road and footway condition can be a major barrier to walking for older people to access bus stops. Earlier studies found that road crossings and bus stop facilities are amongst the main factors deterring older people from using public transport (Marsden et al. 2007; Koffman et al. 2010). From an older person’s perspective, the bus stop approach should be well-maintained, have a level or low gradient, a good road crossing facility and a lower traffic speed.

Recognising the importance of accessibility, various guidelines and best practices have been developed to improve accessibility (e.g. European Bus Directive (EC 2001), ECMT good practice (ECMT 2006), TCRP report 82 (TRB 2002) and PT Access (2008)). These documents cover various aspects of accessibility including bus, bus stop and bus stop approaches. As a result, a substantial effort has been made to improve accessibility to public transport in Europe (EC 1995). Low floor buses (without steps in the bus) have been implemented in Europe since the beginning of the 1990s and are in service almost everywhere in Western and Central Europe. In addition to low floor, buses with a kneeling facility (to reduce the height of the access and buses) and the provision of a ramp have been introduced to enable wheelchair access. Other important features available in modern buses include: Spaces for wheelchairs, bright colour contrasting handrails (to help people with visual impairment to identify support quickly) and designated priority seats (to help people to travel comfortably and without the fear of a trip and fall). In terms of the bus stops and approaches, there are guidelines (TfL 2006) and best practices UNDP (2010) available to improve the accessibility of bus stops. Similarly, ECMT (2006) guidelines cover various aspects of pedestrian environment such as junctions, pedestrian crossings, use of tactile surfaces, etc.

### Affordability

Affordability is an important issue for many older people as they probably have less disposable income in retirement. This means that they are less likely to own their own vehicle and have an increased reliance on public transport (Smith et al. 2006). With limited resources, the cost of travelling could be a major barrier for many old people to travel as often as they would like. In extreme cases, some may be unable to access basic and necessary facilities (hospitals, supermarkets, pharmacies, etc.) not otherwise reachable by walking. With regards to affordability of public transport, older peoples’ needs include: Provision of concessionary fares, ease of use, transferable/flexible tickets and simple fare structure. Fare subsidy is a policy approach often taken to encourage older people to use public transport. The provision of fare reductions for older people is widespread on public transport at both the national as well as local level provided by national/local governments as well as by operators themselves. The scale and the level of concession provided varies from place to place and from country to country. For example in England the concessionary fare scheme (colloquially known is the ‘free bus pass’ for the elderly) allows people over the retirement age for women to enjoy free bus travel throughout the UK, subject to some minor restrictions (e.g. not available in morning peak periods).

### Availability

Availability of public transport within the reach of people’s homes and destinations, with service times and frequencies meeting their requirements, is essential where older people are dependent on bus travel. Of course, it is difficult to provide a regular/frequent service to an area where the demand is low. In many rural areas and small towns, it may not be economically viable to provide a ‘normal’ bus service, but demand responsive transport (DRT) could be a solution to facilitate older people’s travel. (DRT can be described as an advanced, user-oriented form of public transport characterised by flexible routing and scheduling of small/medium vehicles operating in shared-ride mode between pick-up and drop-off locations according to passengers’ needs). In addition, special transport services may be required if the user has reduced physical ability. Recognising this issue, supplementary services can be offered to older people without access to private transport. Examples in the UK of such special transport services include: West Yorkshire’s AccessBus (pteg 2010); Tyne and Wear’s ‘Nexus’. ‘Shopmobility’ can also offer connection between shopping centres and PT stops using various mobility aids including electric scooters, walking frames, etc.

### Acceptability

Older people are sometimes reluctant to take public transport not only because of changes in their physical health, but also because of the challenges that public transport poses to them. Older people who have used public transport throughout their lives are usually more open to considering the various transport alternatives than people who have always used the car as their main mode of transport, who perceive public transport as inconvenient and complicated due to their lack of experience with it (Adler and Rottunda 2006). Older people need to know what public transport services are available, their accessibility and areas served. Acceptability covers a wide range of issues including accessibility, safety, driver attitude (courteous and helpful) and information.

Safety is a serious concern for older people as they are likely to be more severely injured, take longer to recover and suffer greater psychological impact than a younger person in a similar incident. In many cases, older people worry about their safety and are reluctant to take public transport due to factors such as fear of crime, or falling over and becoming injured. For some older people in some countries there can also be a social stigma attached to public transport which may deter them from using it. Such concerns could be addressed by travel training schemes which can help install the confidence and skills in individuals needed to travel on public transport. This may be particularly important for older people who have given up driving due to health reasons and are embarking on the use of public transport for the first time or following a long period of absence. Good practice examples in this area include: UK DfT’s good practice guide for travel training (DfT 2011), “Bus Buddying” scheme in Leeds and CityBee scheme in Barcelona (ptaccess 2008).

Clear, concise, accurate and timely traveller information is useful for all older people regardless of their physical restrictions. However, the level of usefulness relates to the impairment of the traveller. Information, especially regarding the accessibility of buses (vehicle, bus stop) is crucial to older people with mobility problems. Again, the information needs could differ from person-to-person depending on their ability to extract information and their route choice behaviour. Some older people are capable of getting information using recent technologies (e.g. Smartphones) whereas some rely only on printed information: Some may be prepared to spend more time on one bus rather than making a journey needing change (SU 2007). Amongst various ways of providing information, only a few of them are focussed on older people, and information provision is not consistent across Europe. The various forms of information provision useful for older people include: Printed booklets (very useful for those not using latest technology), real time information at bus stops (helping remove uncertainty), on board “Next bus stop” announcement and displays (helping people with visual impairment as well as those with hearing difficulties) and web based services (providing individual door-to-door journey information).

Current information services sometimes fail to adequately meet the needs of older people, as only a few prefer to use technological devices for trip planning or on-trip. A survey among more than 200 older people above 75 years in Austria showed that the vast majority of this group rely on information they retrieve by asking family and friends (40 %) and/or other passengers (20 %). Besides consulting other people, many older people prefer printed information like maps (20 %) or printed timetable booklets (10 %). Technological services like the internet, smartphone apps or other navigation systems are only used by about 5 % of this group (EGALITEplus 2011).

Driver attitude and driving behaviour are additional factors influencing older people’s acceptance of public transport as a mode of transport. Rickert (2009) pointed out that driving behaviour which raises safety concerns is a factor preventing many people from using public transport. In addition, some older people who have previously relied mostly on their car can find it very difficult to make the transition from driving to using public transport. Activities such as travel training and mobility days could be useful for these people to make the transition to public transport. The “Mobility day” event in Salzburg (Austria) is an example of such a scheme which includes an exhibition of products related to the mobility of older people; presentations, brochures and guided tours of public transport; and exchange of views between older people and staff.

Based on the above review, the general requirements of public transport from the perspective of older people are summarised in Table 1.

**Table 1:** General requirements of public transport for older people - taking bus as the example (see GOAL D4.1., 2012, and the references therein)

Issue		Requirements
Accessibility	Bus	Low floor buses Kneeling facility Handrail Priority seating Wheelchair space
	Bus stop	Information (preferably real time information) Audible announcement facility for visually impaired person) Visible and well lit location Clean and protection from rain and sun Provision of seating facilities
	Bus stop approach	Well maintained of footpath to bus stop Level or low gradient footpath Good crossing facility (Signalled crossing, kerbed island at crossing) Lower traffic speed
Affordability		Provision of concessionary fares Ease of use Transferable and flexible tickets Simple fare structure
Availability		Services connecting residence to place of interest (e.g. shopping centre) Demand responsive transport
Acceptability	Safety	Safe approaches to bus stop Safe bus stop location Priority seats Provision of grab rails
	Information	Provision of visual and audio announcement at bus stop Provision of route information displayed at bus stop Use of large font and sufficient details Provision of help point Easily understandable timetable
	Driver attitude	Good attitudes towards older people Driving behaviour (e.g. pulling close to the kerb and smooth acceleration/deceleration) Helpful and informative
	Transition support	Travel information Travel training Travel awareness

The extent to which these requirements are met in reality varies considerably across different European Member States, according to their economic situation, investment in public transport, etc.

## Analysis of Requirements and Current Provision

Recognising the needs of the growing population of older people, various initiatives have been taken at national as well as EU level to improve accessibility and affordability as reported in the earlier sections. However, these measures often attempt to address the requirements considering older people as a homogeneous cohort. In fact, there are differences among older people themselves in terms of their physical abilities or living situations who hence have different requirements of public transport. To analyse these issues in more depth, the cohort of older people was divided into five different profiles in the GOAL project on the basis of different levels of health and mobility. These profiles, are more fully described in Mandl et al. 2013). These five profiles are used as the basis for analysing the requirements in more detail.

### Public Transport Needs of GOAL Profiles of Older People

Identification of common sets of characteristics in the older population in Europe has been based on a cross-national panel database comprising data on health, socio-economic status and social and family networks of more than 55,000 individuals aged 50 or over: This is the SHARE database (Survey of Health, Ageing and Retirement in Europe - http://www.share-project.org). Currently the database contains data collected in 20 European countries; in the course of the GOAL project data from waves 1 and 2 covering 15 countries was used, some countries comprising different language groups^2^. In GOAL, the considered age span of older people was intentionally kept broad by starting at a relatively young age (50). As “age” as such does not provide the largest explanatory power regarding mobility needs, it is important to cover potential mobility limitations which can affect also the “younger” old. Retirement and the accompanying loss of certain social contacts can lead to dissatisfaction and decreasing outdoor activities for some older people, which on the other hand lead to physical degeneration (Pinquart and Schindler 2007).

**Fig. 1:**
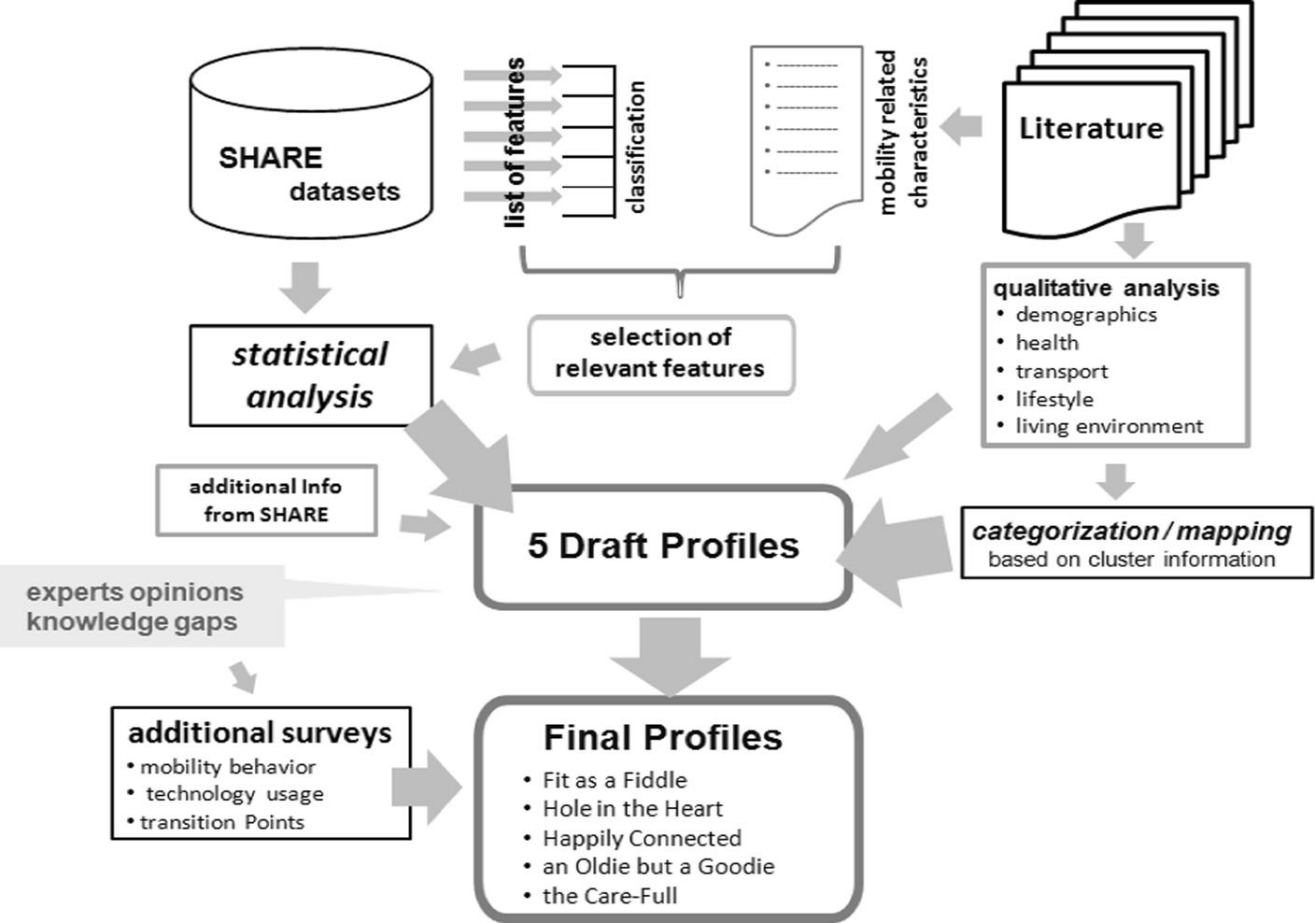
Elaboration process of the profiles of older people

To develop the profiles, descriptive variables related to health and mobility were selected in the database in a first step for eliminating redundancies using a three-dimensional homogeneity analysis (HOMALS, Gifi 1990). Subsequently, the profiles were derived from statistical *k*-means cluster analysis based on the principal components identified in the previous step (mainly features describing demographics and health). The clusters where further developed by linking relevant information concerning physical and mental barriers, regional and socio-demographic differences, transport, life satisfaction and living environment from more than 70 relevant international publications, studies and reports. Relevant aspects which were not covered by the database or the information from the literature were addressed in two small-scale trans-national surveys (among older people and experts and intermediaries working with older people) as well as in the course of two workshops where the profiles at different stages of their elaboration were discussed (see Fig. 1 for the elaboration process of the profiles).

**Fig. 2:**
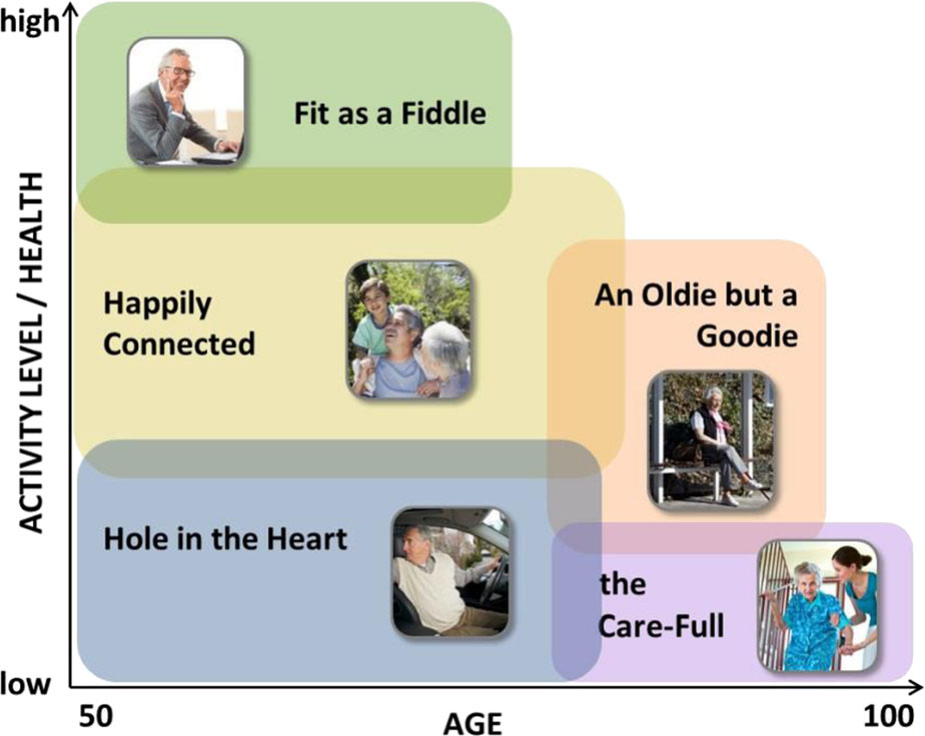
Age and activity level of the profiles of older people (GOAL D2.1, 2012)

Figure 2 illustrates the resulting profiles in relation to two substantial characteristics: Predominant range of age and level of activity. The first group ‘Fit as a Fiddle’ can be described as the youngest and the most active group, while in contrast ‘Hole in the Heart’ and ‘the Care-Full’ are least active profiles due to their health condition. The proportion of the five different profiles (Fit as a Fiddle, Hole in the Heart, Happily Connected, An Oldie but a Goodie and Care-Full) in the database were 37, 13, 32, 13 and 5 %, respectively. Table 2 juxtaposes the profiles according to their main characteristics which have been extracted from data analysis and the literature.

**Table 2:** Summarizing Overview of the Profiles of Older People (Mandl et al. 2013)

		Fit as a Fiddle	an Oldie but a Goodie	Hole in the Heart	The Car-Full	Happily Connected
Demographics	main age group	50-59	80-90	50-75	85-100	60-75
	financial resources	+++	+	---	--	++
	still employed	+++	--	-	---	+
	household information	married or in partnership	single	o	single	married or in partnership
Health	general health	+++	+	--	---	++
	eyesight and hearing	+++	--	o	---	++
	limitation in activities	---	+	++	+++	-
	suffer from pain	---	-	++	+++	--
	dementia / Alzheimer’s	---	+	--	+++	--
	drugs needed	---	+	+++	+++	+
	mobility aid needed	---	+++	o	+++	-
Transport	importance of driving	+++	+	++	+	++ particularly for men
	importance of public transport	--	++	--	-	+ particularly for women
	importance of walking	-	+++	--	+	++
	number and length of trips	+++	--	--	---	++
	purpose of trips	work, leisure, socializing (no different to average society)	socializing, shopping religious services	many trips to hospitals, medical facilities	many trips to hospitals, medical facilities, religious services	entertainment, recreation, sport, socializing, clubs, family, relatives
Environment	problems with infrastructure barriers	---	++	++	+++	-
	afraid of assault / crime	---	+	++	++	-
Life Satisfaction	satisfaction and mental health	+++	++	---	---	+++
	social networks (friends, neighbours, family,…)	++	++	- (family mainly)	-- (family only)	+++
	activities (clubs, volunteering, religious organisations,…)	+++	+	--	---	++
	independency	+++	+	-	---	++
	technology usage	++	--	o	---	+
transitions	live changing events	retirement, birth of grandchildred, severe illness	severe illness, death of a close person	severe illness, loss of social contacts, death of partner	illness, need for (nursing) care, loss of social contact	death of partner, severe illness
	follow-up profiles	Happily Connected, Hole in the Heart	the Care-Full	the Care-Full, an Oldie but a Goodie	x	an Oldie but o Goodie, Hole in the Heart, the Care-Full

(+++ above average; --- below average; o not clear) ratings derived from data analysis, literature, surveys, expert input

The analysis of the issues, current provisions and knowledge gap for each GOAL profile are described below.

#### Fit as a Fiddle

The younger and fit older people belong to this group, most are between 50 and 60 and are married or live in a partnership and/or with their children, have excellent physical and mental health and are still employed. The group members have a comparatively high income and are satisfied with their autonomy and quality of life. This group of older people do not have many problems with technology usage (internet, navigation systems, route planners are used regularly) and often use car as a main mode of transport. They will use public transport only when they give up driving or for specific trips where public transport is clearly the better option. For such people, accessibility, affordability and availability are not issues at present. Having used car as the main mode of transport, people in this group use public transport very little and find it quite difficult to make the transition to public transport. Some have a high perceived risk of using public transport. People in this group are mostly able to use recent technologies and these (e.g. smartphone apps) could be useful in providing public transport information.

There is very little knowledge about the various causes of transition and the best way of supporting the transition to public transport (depending on the causes of transition). Despite having a big pool of smartphone applications, a knowledge gap exists about the requirements of such applications from the prospect of older people (of this profile).

#### Hole in the Heart

Members of this group, in the 50–75 year old age range, suffer from pain and illness or isolation and this severely limits their activities. The car is the preferred mode of transport but when they are not able to drive, they use public transport. Because of their health problems and their mental condition, they make fewer and shorter trips; more trips to hospitals and medical facilities; and need barrier-free infrastructure. Accessibility is an issue for people in this group (due to their poor health) which could be improved on the basis of various best practices and guidelines available. However, macro-economic justification is normally needed for implementation, i.e. economic returns of e.g. increased quality of life and better health due to more trips and participation in social activities. Despite not being a keen public transport user, transition from car to public transport could be suddenly necessary for people in this group, with their reluctance to use public transport also compounded by their safety concerns (real/perceived).

Within the context of economic justification, there is insufficient knowledge about the economic benefits of the quality of life experienced by the older people. In addition, as in the earlier profile, there is very little knowledge about the various causes of transition and the best way to support the transition to public transport.

#### Happily Connected

Most of the group members of this profile are between 60 and 75, are married or live in a partnership. This group has a very active social life and car is the most important transport mode. The usage of technology is high among this profile compared to the other groups, but there are differences within the group. This group of people will use public transport only after giving up driving. However, perceived risks of using public transport may put them off using it. Since they are not regular public transport users, help to make a transition from private car to public transport is crucial for them.

The knowledge gaps in these areas are similar to that for the ‘Fit as a Fiddle’ profile. There is a knowledge gap about the support needed for different groups of people to make the transition to public transport.

#### An Oldie but a Goodie

The members of this group are aged 80 to 90. Most of them are female and are living alone. Despite their age, they are quite healthy and they are not severely limited in activities. Walking and public transport are their preferred modes of transport as the number of car drivers in this group is low. Members of this group avoid technologies, extreme weather, long waiting times, specific social groups and unknown trips (when possible) - and they need good access to public transport. Another major issue for them is safety (actual and perceived) of using public transport. People in this group are generally not users of new technology, so information is needed in terms of the simple and common format such as booklets, phone and radio or trained staff.

Even though there are best practice examples and various guidelines available, there is not enough knowledge about the economic benefits of the quality of life experienced by the older people with accessible transport measures.

#### The Care-Full

This is the group of the very old and frail elderly, who suffer from severe physical and mental diseases such as dementia and develop a very cautious and dependent attitude. Most members of this group depend on care, assistance and the help of others. The members of this group do not leave their homes very often and when they do so, most of them are passengers in a family car or use special transport services. They find travelling (especially in public transport) stressful. They try to avoid new technologies and use familiar technological information sources like TV or radio. Accessibility and availability are key issues. Special transport services (e.g. demand responsive, shared taxi) may be needed to travel around. However, in this tight economic climate, economic justification could be needed to implement such accessible transport measures. For this purpose, further knowledge is needed about the economic benefits of the quality of life experienced by the older people as a result.

Table 3 now summarises a matrix of transport needs/issues according to the five profiles of older people presented here.

**Table 3:** Summary of transport issues of different GOAL profiles

Issue		Fit as a Fiddle	Hole in the Heart	Happily Connected	An Oldie but a Goodie	Care-Full
Accessibility	X	Health related	X	✓	✓	
Affordability	X	✓	X	✓	✓	
Availability	X	X	X	X	✓	
Acceptability	Safety	✓	✓	Y	✓	X
	Driver attitude	X	✓	X	✓	
	Information	X	✓	X	✓	
	Transition support	✓	✓	✓	X	

✓ – Relevant issue, X – Less relevant

Table 3 shows that ‘accessibility’ is less of an issue for ‘Fit as a Fiddle’ and ‘Happily Connected’ groups. This is more of an issue for ‘Hole in the heart’, ‘An Oldie but a Goodie’ and ‘Care-Full’ because of their health condition. There are examples of good practices in Europe addressing accessibility requirements but further research is needed to develop the methodology to justify the implementation of such measures on economic grounds. Affordability is an issue for ‘Hole in the Heart’ and ‘older’ profiles (‘Oldie but a Goodie’ and ‘Care-Full’) who depend on their pension and savings. ‘Availability’ is the issue for the ‘Care-Full’ who rely on the non-conventional transport facilities targeted at them. There are many good practices in these areas depending on the funding availability. Within acceptability, perceived risk of using buses (safety) is an issue for all groups except ‘Care-Full’ who do not use normal public transport. Driver attitude and information provision in high-tech media are issues for ‘Hole in the heart’ and ‘An Oldie but a Goodie’ because of their dependency on public transport. In contrast, ‘transition support’ is an issue for the groups habitual for more driving and living an active life - ‘Fit as a Fiddle’ and ‘Happily Connected’. In relation to addressing these issues, further research needs are discussed in the next section.

## Discussion

Research in the GOAL project has shown that, despite various initiatives to address older people’s public transport requirements, there are some gaps in the understanding of some related issues. These issues need to be addressed to widen the access to public transport for the ever increasing population of older people.

### Research on the Issues of Transition to Public Transport

Some older people are not regular public transport users. However, with age related or other health issues, they may have to make transitions from private cars to public transport. The experience of transition may differ from person to person and depends on the causes of transition. There is a knowledge gap of the reasons of transition and consequences to a person making such transition. It would be necessary to identify at which point in time (e.g. at retirement or poor health condition) and in which way (e.g. incentives, specific support and awareness campaign) training could be given so that older people become familiarised with the mobility alternatives available. This should increase their skills and willingness to use public transport and makes it easier to use when needed (e.g. when they change to the HH group due to illness). In this respect, research is needed to develop methodologies to implement targeted intervention based on the prior experience of a person making transition to public transport. Such intervention could be simply a provision of published information or a guided tour of a journey an older person is planning to make.

### Methodology for Assessing Benefits of Accessibility Measures

Accessibility has been the main focus of initiatives to help older people use public transport. As a result, there are a number of best practice examples and various guidelines for the implementation of accessible facilities. For example, low floor buses and provision of space for wheelchair in a bus. This may involve improvements to the walking experience to/from bus stops, enhanced facilities at bus stops (shelters, build-outs, real-time information provision, etc.) and improvements to the bus itself (low floor, wheelchair accessible, coloured handrails for the visually impaired, etc.) However, in the current economic climate, economic justification is particularly needed for such measures. At this stage there is no defined methodology to assess the economic benefits of such measures in terms of improved quality of life (rather than traditional economic value of time savings only). Inclusion of such benefits will help justifying the implementation of the accessibility measure.

### Safety and Security Concern of Using Public Transport

Older people take longer to recover from injuries than younger people and the results could affect their life experience for some time. Hence, safety (actual and perceived) is a major concern for many older people using public transport. There are good practices available to reduce such safety concerns (e.g. travel training). However, there is no clear identification of the level of perceived and actual safety concerns of older people and the best ways of mitigating them. One such topic is trips or falls in buses in relation to the driving behaviour (e.g. speed, acceleration and deceleration). Such driving behaviour data could be collected from automatic vehicle location (AVL) systems.

Security, either on the way to the bus stop or on-board, is also a major issue and older people are often more afraid of insecure environments than unsafe ones. Ensuring a secure environment helps older people to more easily make a transition to public transport. The authorities and operators could play an important role to increase both the effectiveness of measures to improve security in real terms and to transmit that message to older people and their relatives.

## Conclusions

Public transport is important to older people’s quality of life, their sense of freedom and independence. Access to public transport can help older people to avail themselves of goods, services, employment and other activities. In the ideal world, older people would like to have accessible, affordable, frequent, comfortable, door-to-door, spontaneous services with access to a large variety of destinations over an extended period of time. In this paper, four main issues (affordability, availability, accessibility and acceptability) have been used as the basis for assessing the public transport needs of five GOAL profiles of older people (Fit as a Fiddle, Hole in the Heart, Happily Connected, An Oldie but a Goodie, the Care-Full).

The literature review showed that, with a growing population of older people, their needs are being addressed or recognised in many national and international policies. As a result, a number of best practices covering various aspects of public transport (especially accessibility issues) are implemented at various local, national and EU levels in different countries around Europe. However, the level of implementation varies from country to country and place to place. The analysis of current provisions against the needs of different GOAL profiles of older people showed some areas of knowledge gaps including: research on the issues of transition to public transport; methodology for assessing benefits of accessibility measures; and safety and security concerns of using public transport. These issues need to be understood and tackled to provide older people with friendly and useable public transport for 21st century.

## Footnotes

1 Additional information on transport policy approaches has been provided by the project TRACY, which reviewed the extent to which national transport policymaking and practice across the EU (and beyond) caters for transport needs of an ageing society (http://cordis.europa.eu/result/rcn/149298_en.html).

2 Austria, Germany, Sweden, Netherlands, Spain (Castilian), Italy, France, Denmark, Greece, Switzerland (German/French/Italian), Belgium (French/Flemish), Israel (Hebrew/Arabic/Russian), Czech Republic, Poland, Ireland
